# Innate immune responses to RNA: sensing and signaling

**DOI:** 10.3389/fimmu.2024.1287940

**Published:** 2024-01-25

**Authors:** Xiaohan Luan, Lei Wang, Guangji Song, Wen Zhou

**Affiliations:** ^1^ Shenzhen Key Laboratory of Biomolecular Assembling and Regulation, Southern University of Science and Technology, Shenzhen, Guangdong, China; ^2^ Department of Immunology and Microbiology, School of Life Sciences, Southern University of Science and Technology, Shenzhen, Guangdong, China; ^3^ Department of Systems Biology, School of Life Sciences, Southern University of Science and Technology, Shenzhen, Guangdong, China

**Keywords:** RNA-sensing pathways, RNA sensors, innate immunity, pattern recognition receptor, RNA vaccines, disease

## Abstract

Nucleic acids are among the most essential PAMPs (pathogen-associated molecular patterns). Animals have evolved numerous sensors to recognize nucleic acids and trigger immune signaling against pathogen replication, cellular stress and cancer. Many sensor proteins (e.g., cGAS, AIM2, and TLR9) recognize the molecular signature of infection or stress and are responsible for the innate immune response to DNA. Remarkably, recent evidence demonstrates that cGAS-like receptors acquire the ability to sense RNA in some forms of life. Compared with the nucleic-acid sensing by cGAS, innate immune responses to RNA are based on various RNA sensors, including RIG-I, MDA5, ADAR1, TLR3/7/8, OAS1, PKR, NLRP1/6, and ZBP1, via a broad-spectrum signaling axis. Importantly, new advances have brought to light the potential clinical application of targeting these signaling pathways. Here, we highlight the latest discoveries in the field. We also summarize the activation and regulatory mechanisms of RNA-sensing signaling. In addition, we discuss how RNA sensing is tightly controlled in cells and why the disruption of immune homeostasis is linked to disease.

## Introduction

Single- or double-stranded RNA is used as the genetic material for RNA viruses. To sense viral infection, animals encode a set of PRRs (pattern recognition receptors), involving several RNA sensors that directly recognize viral RNA and trigger immune responses (1). RNA sensors exhibit divergent activation mechanisms; for example, some detect ssRNA (single-stranded RNA), such as TLR7/8 (toll-like receptor 7/8); and several sense dsRNAs (double-stranded RNAs), such as RNA helicase RIG-I (retinoic acid-inducible gene I), MDA5 (melanoma differentiation-associated protein 5), TLR3, PKR (protein kinase R), and OASes (oligoadenylate synthases) (2–5). In addition to RNA types, an important feature of RNA sensors is their sensitivity to RNA length. For example, RIG-I is specifically activated by short dsRNAs with specific features (5′ diphosphate/5′-triphosphate dsRNA, duplex structure, lacking ribose 2′-O-methylation), while MDA5 does not require these triphosphate ends of RNA; instead, it prefers to sense longer dsRNAs (typically >500 bp in length) (6, 7). Collectively, these sensors detect both exogenous and endogenous RNA and play critical roles in viral infection, cellular stress, RNA metabolism, and immune homeostasis (**Table 1**).

**Table 1:** RNA sensing and signaling.

Sensor	Ligand	Adaptor/ Effector	Transcription factor	Immune responses	Disease relevance	Reference
RIG-I	short, duplex structure, lacking ribose 2′-O-methylation, 5′-pp/5′-ppp dsRNA (<0.5 kb)	MAVS	IRF3, IRF7, NF-κB	Type I IFNs, inflammatory cytokines expression	Singleton -Merten syndrome-2	(7–10)
MDA5	long dsRNA (>0.5 kb)	MAVS	IRF3, IRF7, NF-κB	Type I IFNs, inflammatory cytokines expression	Aicardi-Goutières syndrome (AGS); Type 1 diabetes mellitus 19; Singleton-Merten syndrome-1; Immunodeficiency-95	(9, 11–16)
TLR3	long dsRNA (≥90 bp)	TRIF	IRF3, NF-κB	Type I IFNs, inflammatory cytokines expression	Life-threatening COVID-19 pneumonia; Immunodeficiency-83	(17–20)
TLR7	uridine-containing ssRNA and guanosine	MyD88	IRF7, NF-κB	Type I IFNs, inflammatory cytokines expression	Life-threatening COVID-19 pneumonia; Systemic lupus erythematosus 17	(17, 21–23)
TLR8	short ssRNA and uridine	MyD88	IRF7, NF-κB	Type I IFNs, inflammatory cytokines expression	Immunodeficiency-98 with autoinflammation	(17, 24–26)
OAS1	dsRNA (≥18 bp)	RNase L	N/A	ssRNA (e.g., ribosome RNA) degradation	Life-threatening COVID-19 pneumonia; Immunodeficiency-100	(27–31)
PKR	dsRNA (≥30 bp)	eIF2α	N/A	Translation initiation and protein synthesis arrestation	Neurodevelopmental syndrome	(32, 33)
NLRP1	dsRNA (>500 bp)	Caspase-1	N/A	Inflammasome, Pyroptosis	N/A	(34)
NLRP6	dsRNA	Caspase-1	N/A	Inflammasome, Pyroptosis	N/A	(35)
ZBP1	Z-DNA, Z-RNA	RIPK1, RIPK3	N/A	PANoptosis	IAV infection; cardiotoxicity	(36–38)
cGLR1 (Fly cGAS)	dsRNA	STING	Unknown	Antiviral immunity	N/A	(39, 40)
cGLR2 (Fly cGAS)	dsRNA	STING	Unknown	Antiviral immunity	N/A	(41)
CdnE03 (Bacteria cGAS)	A specific, structured, viral-derived RNA	Cap15	N/A	Antiviral CBASS immunity	N/A	(42)

N/A, not available.

cGAS (cyclic GMP-AMP synthase) is one of the most important dsDNA (double-stranded DNA) sensors (43). DNA is located mainly in the nucleus and mitochondria. DNA sensors are activated mainly in the cytoplasm to prevent autoreactivity to self-DNA. The observation of cGAS in the nucleus led to the striking discovery that cGAS is kept inactive through tight binding to chromatin (44–51). Distinct from DNA, RNA is abundant in the cytosol, where many RNA sensors are located and activated. The self-/nonself-discrimination mechanism is necessary and complex; for example, some viral RNAs bear a 5′-ppp, a signal that drives the activation of RIG-I-mediated antiviral immunity (6). Similarly, ADAR1 (adenosine deaminase acting on RNA 1) catalyzes the hydrolytic deamination of adenosine to inosine (A-to-I editing) within dsRNA, preventing unwanted immune activation by MDA5 (52, 53). Notably, the evolution of technologies and principles led to the discovery of additional RNA sensors and the identification of phase separation as a novel regulatory mechanism in RNA sensing (34, 35, 39, 40, 54).

In this review, we discuss several crucial RNA-sensing pathways involved in antiviral and antitumor immunity. We focus on the structural and biochemical mechanisms of their activation and regulation, discussing how multiple immune signaling pathways sense RNA diversity, and newly emerging questions that remain unanswered in the field.

## Multiple immune signaling pathways sense RNA diversity

**Figure 1:**
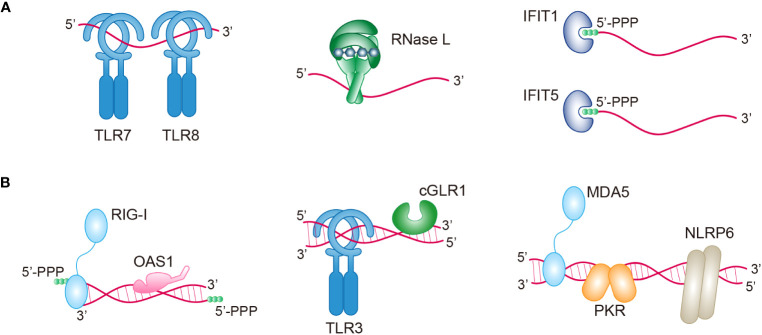
Multiple immune sensors/receptors in RNA-sensing signaling pathways. An overview of the sensors/receptors recognizing ssRNAs **(A)** or dsRNAs **(B)** in antiviral immunity. Upon RNA binding, these proteins can directly act on RNA (RNase L, IFIT1, IFIT5, OAS1, and PKR), or they can indirectly activate various pathways (TLR7, TLR8, RIG-1, TLR3, cGLR1, MDA5, and NLRP6). TLR7/8/3, toll-like receptor 7/8/3; RNase L, ribonuclease L; IFIT1/5, IFN-induced protein with tetratricopeptide repeats -1/5; PPP, triphosphate; RIG-I, retinoic acid-inducible gene I; OAS1, oligoadenylate synthase 1; cGLR1, cGAS-like receptor 1; MDA5, melanoma differentiation-associated gene 5; PKR, protein kinase R; NLRP6, NOD-like receptor family pyrin domain containing 6.

RNA is typically a single-stranded biopolymer that is present in almost all living organisms. In addition to being involved in diverse cellular processes, RNA can also function as an immunostimulant that triggers cellular immune responses (55). Across various life forms, from bacteria to vertebrates, multiple signaling mechanisms have evolved to detect the presence of foreign RNA. Among these, ssRNAs are recognized by TLR7 and TLR8 (56). ssRNA is also a substrate for RNase L (ribonuclease L), which is an immune nuclease when upstream OAS signaling is activated (57). IFIT1 and IFIT5 (interferon-induced proteins with tetratricopeptide repeats 1 and 5, respectively) can bind the 5′-triphosphaste ends of ssRNAs to inhibit RNA translation and restrict viral replication (58) (**Figure 1A**).

However, the presence of self-complementary sequences in RNA sometimes causes the formation of double-stranded structures. The presence of dsRNA in cells is normally related to viral infection. For example, cellular dsRNA is derived from dsRNA viruses, positive-strand RNA viruses, negative-strand RNA viruses, and even DNA viruses (59). Importantly, a recent study has demonstrated the presence of endogenous dsRNAs, which can be produced during normal physiological processes and disease processes (59). Many receptors, including RIG-I, OAS1, TLR3, cGLR1 (cGAS-like receptor 1), MDA5, PKR, and NLRP6 (nucleotide-binding domain and leucine-rich repeat pyrin domain containing protein 6), have evolved in animals to directly recognize both pathogen-derived and host-derived dsRNA (**Figure 1B**). Interestingly, although these dsRNA sensors share a common feature of dsRNA binding, each sensor exhibits a preference for distinct types of dsRNA based on factors such as RNA length, sequence, end structures, and chemical modifications. The diversity of RNAs serves as a unique signature for different sensors and can be utilized to coordinate complex immune signaling networks (**Table 1**). The specific signaling mechanisms of RNA sensing are discussed in detail below.

## Pathogen sensing, signaling and regulation of the RLR-MAVS pathway

**Figure 2:**
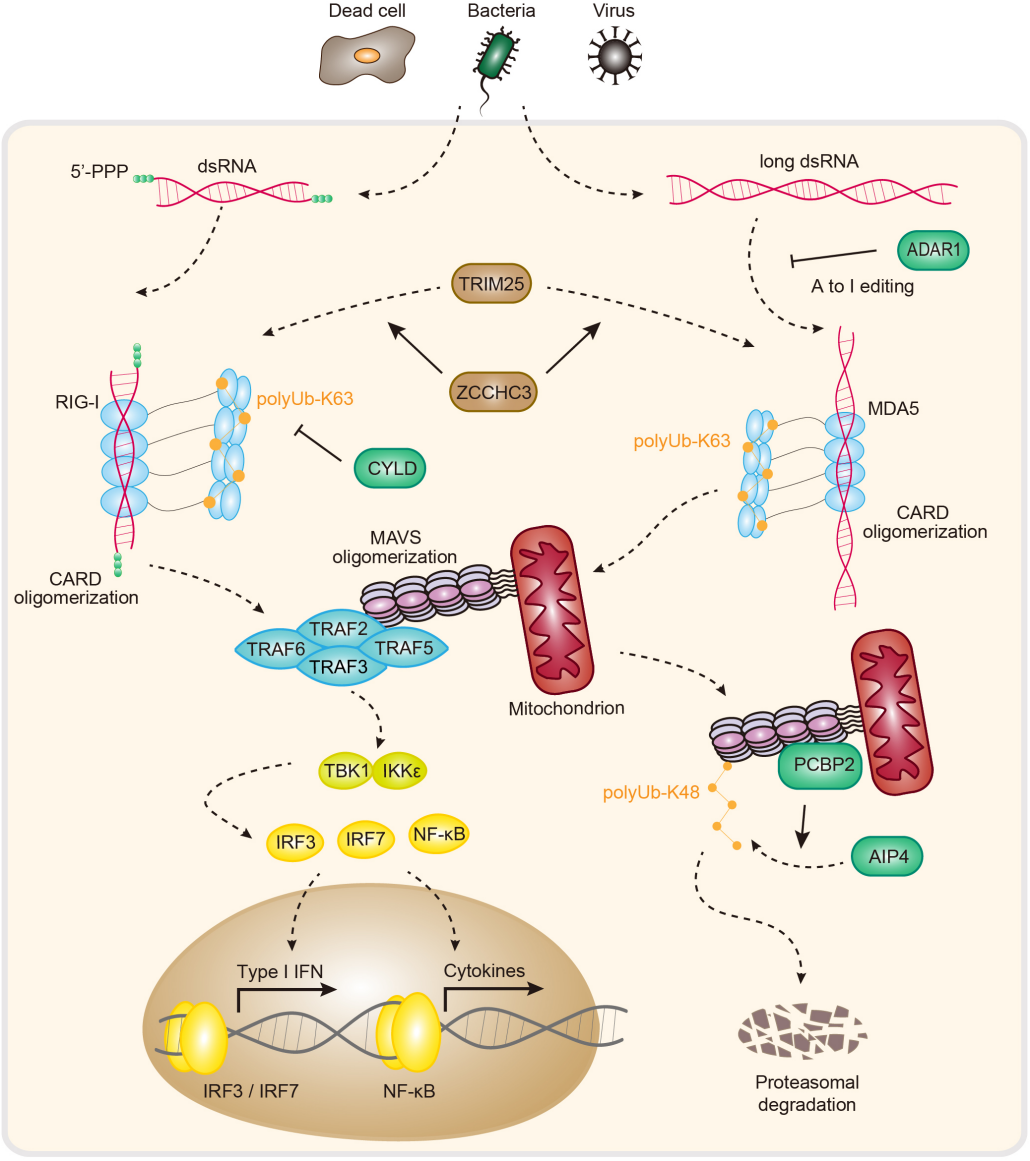
Pathogen sensing, signaling and regulation of the RIG-I and MDA5 pathways. In animals, RIG-I and MDA5 directly detect RNA molecules associated with pathogen infection. The 5′-ppp of dsRNA activates RIG-I, while long dsRNA activates MDA5. The RNA-induced formation of filament structures further recruits MAVS via homotypic CARD-CARD interactions. This process causes MAVS oligomerization and triggers type I IFN-/NF-κB-dependent antiviral immune responses. ADAR1, adenosine deaminase acting on RNA 1; TRIM25, tripartite motif containing 25; ZCCHC3, zinc finger CCHC-type containing 3; RIG-I, retinoic acid-inducible gene I; Ub, ubiquitin; CARD, caspase recruitment domain; CYLD, CYLD lysine 63 deubiquitinase; MDA5, melanoma differentiation-associated gene 5; MAVS, mitochondrial antiviral signaling protein; TRAF 2/3/5/6, tumor necrosis factor receptor-associated factor 2/3/5/6; TBK1, TANK-binding kinase 1; IKKϵ, IκB kinase ϵ; IRF3/7, interferon regulatory factor 3/7; NF-κB, nuclear factor-κB; PCBP2, poly C−binding protein 2; AIP4, atrophin-1-interacting protein 4; IFN, interferon.

RLRs (RIG-I-like receptors) are RNA sensors that include three members: RIG-I, MDA5 and LGP2 (laboratory of genetics and physiology 2) (2, 60). All RLRs are located mainly in the cytosol and contain a central helicase domain and a CTD (carboxy-terminal domain). RIG-I and MDA5 additionally harbor two CARDs (caspase activation and recruitment domains) (60, 61) (**Figure 2**). The helicase domain and CTD contribute to RNA binding (62), and the CARD mediates downstream signal transduction via autooligomerization and further recruitment of MAVS (mitochondrial antiviral signaling protein). LGP2 is widely believed to be a regulator of RIG-I and MDA5 owing to the lack of a CARD (60).

Growing evidence suggests that RIG-I plays essential roles in innate antiviral immunity (60). Biochemical and structural studies have elucidated the requirement of dsRNA for RIG-I activation, including the 5′-pp/5′-ppp moiety and an unmethylated 2′-O group of the first nucleotide. This specificity is mainly controlled by the CTD of RIG-I (8, 63). The recognition of 5′-pp/5′-ppp dsRNA confers RIG-I with the advisable strategy for self-/nonself discrimination, as 5′-ppp dsRNA is normally produced during viral replication. The 5′-ppp ends of host RNA are capped with 7-methyl guanosine and sometimes modified with 2′-O-methyl, providing an additional strategy to distinguish self from nonself (6, 64). Recent analysis revealed that RIG-I adopts two different conformations for distinguishing host RNA from viral RNA (62). The cryo-EM structures of RIG-I complexes with viral and host RNA demonstrated that upon viral RNA binding, RIG-I adopts a high-affinity conformation that helps with RIG-I activation and downstream signaling. In contrast, its binding to host RNA preferentially results in the formation of an autoinhibited conformation that facilitates RNA release from the complex. Together, these unique properties of RIG-I RNA recognition ensure selective antiviral sensing and prevent unwanted autoimmunity.

In an inactive state, the CARD interacts with parts of the helicase domain of RIG-I. Upon dsRNA binding to the CTD, stable RIG-I-RNA binding releases the CARD, which then undergoes tetramerization to promote RIG-I-MAVS complex formation via CARD-CARD interactions (9). Upon interacting with RIG-I, MAVS forms higher-order filaments and recruits TRAF2/3/5/6 (tumor necrosis factor receptor-associated factor 2/3/5/6), activating the transcription factors IRF3/7 (interferon regulatory factor 3/7) and NF-κB (nuclear factor-κB), and inducing type I IFN (interferon) expression and antiviral immunity (65–67) (**Figure 2**).

The innate immune response to RNA via RIG-I needs to be tightly controlled to allow potent signal activation while maintaining immune homeostasis under physiological conditions. As such, inappropriate RIG-I activation is a direct cause of severe autoimmune disorders (68). Several mechanisms have been reported to control the immune homeostasis of RIG-I signaling. For example, RIG-I activity is controlled by ubiquitination and deubiquitination (**Figure 2**). In brief, TRIM25 (tripartite motif containing 25) polymerizes K63-linked polyubiquitin chains to RIG-I at K172, stabilizing the interaction between RIG-I and MAVS. In addition, ZCCHC3 (zinc finger CCHC-type-containing 3) promotes K63-linked polyubiquitination of RIG-I and MDA5 via TRIM25 to facilitate antiviral RNA responses (69, 70). In contrast to those of TRIM25 and ZCCHC3, the ubiquitin carboxyl-terminal hydrolase CYLD, USP3 (ubiquitin-specific peptidase 3) and USP21 negatively regulate RIG-I-MAVS signaling by removing K63-linked polyubiquitin chains (71–73). In another example of ubiquitination, however, the attachment of K48-linked ubiquitin chains to RIG-I by RNF125 (ring finger protein 125) results in signal termination due to the proteasome-dependent degradation of RIG-I (74). In addition to regulation by ubiquitylating/deubiquitylating enzymes, RIG-I-MAVS signaling is also controlled by interacting proteins (**Figure 2**). For example, PCBP2 (poly(rC)-binding protein 2) interacts with MAVS and recruits the HECT domain–containing E3 ligase AIP4 to attach K48-linked ubiquitin chains to MAVS, inducing proteasomal degradation of MAVS to negatively regulate the RIG-MAVS axis (75). Furthermore, tetherin promotes the K27-linked ubiquitination of MAVS by recruiting MARCH8, enhancing the autophagic degradation of MAVS to negatively regulate RLR-mediated type I IFN signaling (76). In addition to ubiquitination, other PTMs (posttranslational modifications), including phosphorylation, SUMOylation, ISGylation, and acetylation, also play essential roles in regulating the RIG-I-MAVS pathway (77–81). Recent analysis revealed that two SNPs (c.1118A>C [p.Glu373Ala] and c.803G>T [p.Cys268Phe]) in *DDX58* (encoding RIG-I) can increase IFN and ISG expression, causing SGMRT2 (Singleton-Merten syndrome-2) to lead to glaucoma, aortic calcification, and skeletal abnormalities, indicating an important role in autosomal-dominant multisystem disorders (10). Together, these regulatory mechanisms and dysfunction–disease associations help explain the importance of RIG-I–MAVS signaling in immune homeostasis.

## ADAR1 prevents MDA5-driven autoimmunity

Like RIG-I, which recognizes viral infection, MDA5 senses viral dsRNA and recruits the same signaling adaptor, MAVS, to activate antiviral immunity (**Figure 2**). This is not surprising because MDA5 shares the same domain architecture as RIG-I (**Figure 2**). However, given that MDA5 and RIG-I differentially induce type I IFNs in response to different viral pathogens, it is clear that MDA5 can function independently of RIG-I (82). Recent structural and biochemical studies revealed that MDA5 has distinct biochemical properties for dsRNA recognition. Indeed, RIG-I prefers short dsRNA (<0.5 kb) with 5′-ppp ends, while MDA5 prefers long dsRNA (>0.5 kb) and is independent of the end of dsRNA (7). It was reported that the length of dsRNA (1-7 kb) progressively increases the IFN responses mediated by MDA5, suggesting that MDA5 is activated by RNA in a length-dependent manner (11). Taken together, these findings demonstrate that, unlike RIG-I, MDA5 evolved to use a molecular mechanism to sense dsRNA on the basis of length.

However, the detailed mechanism of the length-dependent activation of MDA5 remains unclear. Evidence in the field supports some potential models. One model is the ATP-driven length discrimination of MDA5. Hur et al. proposed that MDA5 activation is dynamically controlled by its CTD-dependent filament assembly and helicase-dependent disassembly. ATP hydrolysis triggers the disassembly of filaments only from their ends, suggesting that, compared with long dsRNA, MDA5 tends to be released from short dsRNAs (83). However, this kinetic mechanism does not fully explain why the stem loops within 3′ untranslated regions of endogenous transcripts (long RNA) do not potently activate MDA5. These findings suggested that self- or self-discrimination is additionally controlled by other factors and await further investigation.

Dysfunction of MDA5 is a direct cause of severe autoimmune disorders. *IFIH1* (encoding MDA5) mutations reportedly result in aberrant type I IFN expression, leading to AGS (Aicardi–Goutières syndrome), an inflammatory disease characterized by cerebral atrophy, leukoencephalopathy, intracranial calcifications, and chilblain skin lesions (12). Several missense mutations in *IFIH1* (such as R337G, D397V, G495R, R720Q, R779C and R779H) enhance MDA5-dsRNA binding affinity and contribute to the excessive production of type I IFNs, also indicating that the strict regulation of MDA5 activity is essential for homeostasis maintenance (13). Relatedly, recent studies surprisingly revealed that defects in A-to-I editing cause MDA5 activation and autoimmunity, linking RNA modification to MDA5 immune signaling. Further studies revealed that ADAR1 is responsible for the control of host RNA sensing partially via MDA5. In support of this model, genetic analysis further demonstrated that ADAR1 deficiency causes MDA5-dependent inflammatory diseases in humans and mouse models (84). Human ADAR1 has two isoforms, p110 and p150. ADAR1-p110 is constitutively expressed and located in the nucleus, and the IFN-inducible isoform ADAR1-p150 is localized both in the nucleus and the cytosol (85). Both isoforms contain a Zβ domain, a dsRNA-binding domain, and a C-terminal deaminase domain, with an additional N-terminal Zα domain occurring only in ADAR-p150 (86). Editing of endogenous RNAs by ADAR1 prevents MDA5-driven long-dsRNA sensing and downstream IFN expression, providing a safety net for maintaining normal homeostasis (52, 53).

## TLR (TLR3, TLR7 and TLR8) signaling pathway

**Figure 3:**
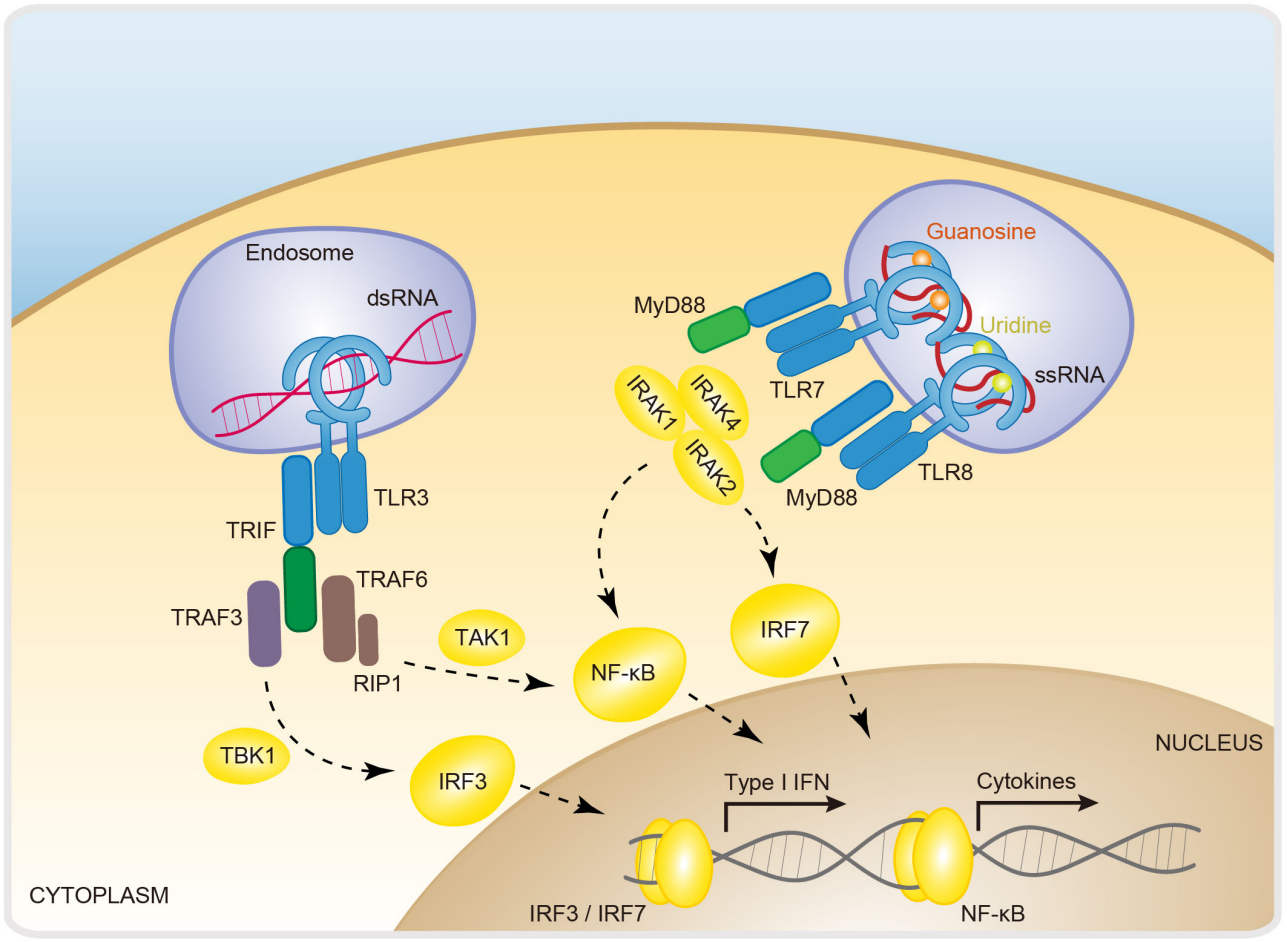
RNA-sensing via Toll-like receptors. Dimerization is a fundamental step in the activation of Toll-like receptors. TLR3, TLR7, and TLR8 are dimerized and activated by the recognition of dsRNA, ssRNA/guanosine, and ssRNA/uridine, respectively. These signals are further activated via the adaptor proteins TRIF for TLR3 and MyD88 for TLR7/8 and the downstream transcription factors IRF3, IRF7 and NF-κB. TLR7/8/3, toll-like receptor 7/8/3; TRIF, TIR domain-containing adapter-inducing interferon-β; TRAF3/6, TNF receptor associated factor 3; RIP1, receptor-interacting protrin1; IRAK 1/2/4, interleukin-1 receptor-associated kinase 1/2/4; MyD88, myeloid differentiation primary response gene 88; IRF3/7, interferon regulatory factor 3/7; NF-κB, nuclear factor-κB; IFN, interferon.

TLRs (toll-like receptors) are the first protein family defined as PRRs (87). They are expressed mainly on immune cells and epithelial cells. Upon sensing diverse PAMPs or DAMPs (danger-associated molecular patterns) (e.g., DNA, RNA, lipopolysaccharide, and flagellin), TLRs recruit different adaptors, including MyD88 (myeloid differentiation primary response gene 88), TRIF (TIR-domain-containing adaptor protein inducing interferon-β), TRAM (TRIF-related adaptor molecule) and TIRAP (Toll/interleukin-1 receptor domain-containing adapter protein), and activate various downstream signaling cascades (e.g., IFNs, cytokines and chemokines) (17, 88, 89). TLRs are composed of three motifs, namely, an LRR (leucine-rich repeat) domain, a TM (transmembrane) domain, and a TIR (Toll/IL-1R) domain (17). The extracellular region LRR, shaped like a horseshoe, is responsible for recognizing PAMPs and DAMPs, and the intracellular domain TIR mediates adaptor recruitment and signal transduction (90). The TLR family contains many members that can be found in endosomes and the cell membrane (17). Among TLRs, TLR3, TLR7, and TLR8 are predominantly localized on the endosome and are responsible for RNA recognition (1). Upon ligand binding, TLRs undergo heterodimerization or homodimerization to initiate signal transduction (21, 91). After dimerization, the TIR domain recruits various downstream adaptor proteins to transduce signals; for example, TRIF is used for TLR3 signal transduction, and MyD88 is an adaptor for TLR7 and TLR8 (92) (**Figure 3**).

TLR3 was identified as a dsRNA sensor that responds to the chemical ligand poly(I:C) (polyinosinic-polycytidylic acid), a miscellaneous synthetic dsRNA molecule (3, 93). TLR3 is capable of triggering IFN and cytokine production during infection with VSV (vesicular stomatitis virus), EMCV (encephalomyocarditis virus), and WNV (West Nile virus), indicating that TLR3 acts as a direct viral sensor (94, 95). The ectodomain of TLR3 has a horseshoe-like shape, providing a convex surface for dsRNA binding. Upon dsRNA binding, TLR3 undergoes dimerization and recruits TRIF, which further recruits TRAF3 and TRAF6. TRAF3 activates the IKK-related kinase TBK1 (TANK-binding kinase 1) and sequentially phosphorylates IRF3. Then, the phosphorylated homodimer IRF3 translocates into the nucleus, where it induces the expression of type I IFNs and ISGs (IFN-stimulated genes) (96). TRAF6 recruits the kinase RIP1 (receptor-interacting protein 1) and subsequently activates TAK1 (TGF-β-activated protein kinase 1) and NF-κB to induce inflammatory cytokine expression (17, 96) (**Figure 3**). The crystal structure demonstrated the formation of a stable complex between the dimeric form of TLR3 and a 46-bp dsRNA. However, the 46-bp dsRNA is not enough to induce a robust cellular immune response and requires long (≥90 bp) dsRNA (18, 97). The cryo-EM structure of TLR3 with a 400 bp long dsRNA revealed that dimeric TLR3 proteins are clustered along long dsRNAs in a highly organized manner. Importantly, the binding surface required for TLR3 clustering is indispensable for robust signal activation, explaining the ability of long dsRNAs to act as active ligands of TLR3 (98). In addition to its canonical antiviral activity, TLR3 was recently reported as a potential mechanism for inducing antitumor immunity, as the expression of TLR3 dramatically increases the expression of IFN-λ1 by cDC1s (conventional type 1 dendritic cells) and promotes tumor-inhibitory CTL (cytotoxic T lymphocyte) responses (99). This seminal finding unleashed an avalanche of interest in TLR signaling, with an initial focus on the potential of druggable targeting of the TLR3 pathway in antitumor therapeutics.

Like that of TLR3, the activation of TLR7 and TLR8 causes an IFN-/NF-κB-dependent immune response. A notable exception is that the activating ligand for TLR7/8 is ssRNA (95) (**Figure 3**). Biochemical and structural studies have revealed a two-step mechanism for the activation of TLR7 and TLR8 (21). For example, TLR7 binds two ligands (ssRNA and guanosine) in a sequential way. First, uridine-containing ssRNAs interact with TLR7 to prime it for guanosine binding, both of which are required for the induction of TLR7 dimerization (21). The TLR7 dimer subsequently recruits MyD88. MyD88 then recruits IRAK4 (IL-1 receptor-associated kinase 4) via death domain homointeraction, after which IRAK1 and IRAK2 are recruited by activated IRAK4 and further undergo autophosphorylation to form Myddosomes, which induce NF-κB and IRF7 activation and subsequent inflammatory cytokine and type I interferon expression (96). Likewise, TLR8 has two binding sites for recognizing two degradation products of ssRNA, uridine and a short oligonucleotide (24). Both binding sites are required to stabilize the TLR8 dimer and are essential for TLR8-MyD88 signaling activation (24). It has been proposed that GU-rich ssRNAs are potent natural ligands for TLR7 and TLR8 activation. Major questions for this model include how are these specific forms of ligands generated? Recently, a pioneering study characterized the lysosomal endoribonuclease RNase T2 as the molecular cue of TLR8-dependent RNA recognition and signaling (100). It was proposed that RNase T2 cleaves ssRNA and generates purine-2',3'-cyclophosphate-terminated oligoribonucleotides and uridine, thereby potently activating TLR8 signaling (100).

Recent studies have shown that some life-threatening COVID-19 cases are caused by monogenic inborn immune errors, such as those involving TLR3 and TLR7 (19, 22). The TLR3 mutation (*TLR3* p.Pro554Ser) is an autosomal LOF (loss-of-function) mutation that causes an impaired TLR3/IRF7-dependent type I IFN response, underlying life-threatening COVID-19 pneumonia (19). Like in TLR3, LOF variants of the X chromosome of TLR7 (*TLR7* p.[Val795Phe]) are also associated with an impaired IFN response (22). In contrast to the reported TLR3 and TLR7 immune-impaired variants, several *TLR8* mutants exhibit excessive activation associated with X-chromosome-linked disease and IMD98 (immunodeficiency 98) with autoinflammation (25).

## The OAS-RNase L pathway in COVID-19 and autoimmune diseases

**Figure 4:**
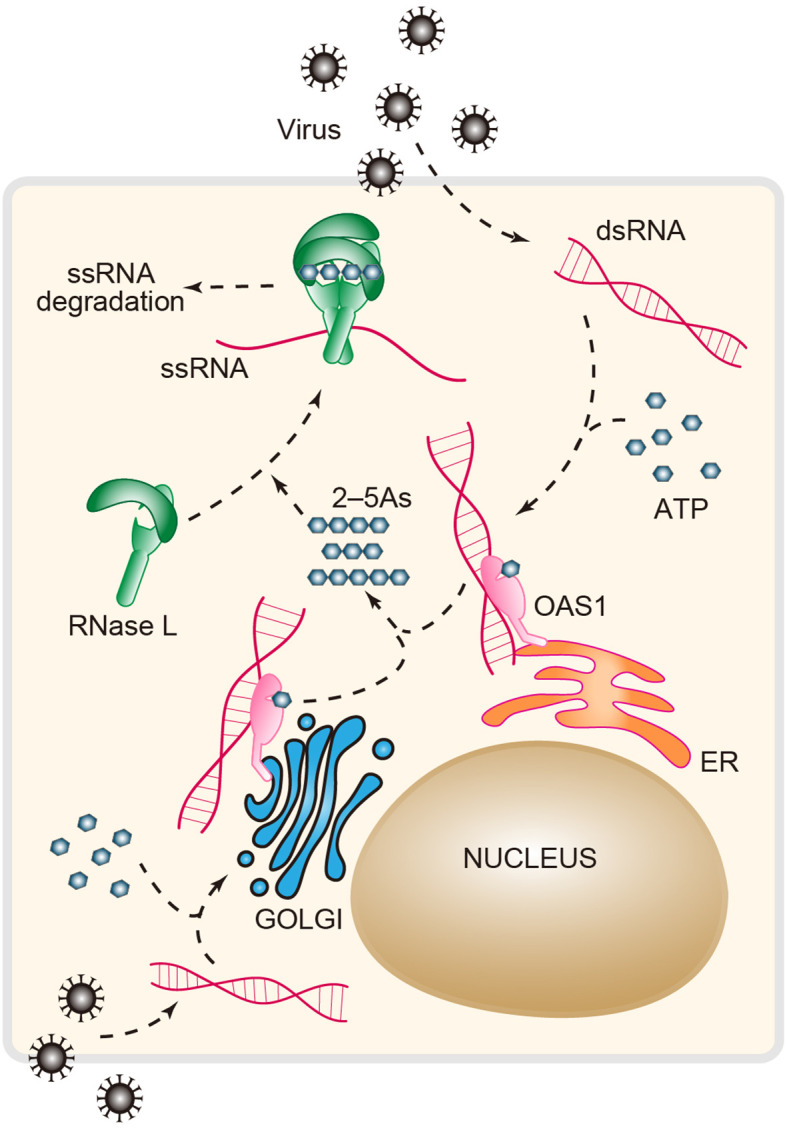
The antiviral OAS-RNase L pathway. Upon dsRNA recognition, OAS1 synthesizes the second messenger 2′–5′-linked oligoadenylates (2–5As, length ranging from 2 to 30) using ATP as a substrate in response to viral infection. 2–5As directly binds the monomer form (inactive) of RNase L and induces its dimerization (active form). The active RNase L then digests ssRNA to limit viral infection. RNase L, ribonuclease L; OAS1, oligoadenylate synthase 1.

OAS1 is a dsRNA sensor that functions as a viral restriction factor. dsRNA enzymatically activates OAS1 to produce linear 2–5As (2′–5′ oligoadenylates) and subsequently activates the downstream effector RNase L to block viral replication (5, 101) (**Figure 4**). The 2′–5′ phosphodiester linkages that occur in 2–5A are rare in biology compared to the common forms of 3′–5′ phosphodiester bonds found in DNA and RNA. Importantly, OAS1 was the first enzyme identified in mammals that produces the native 2′–5′ linkage, followed by the discovery of cGAS-like receptors that synthesize a broad range of products containing 2′–5′ phosphodiester bonds (43, 54, 102–104). Mechanistically, OAS1 binds dsRNA and undergoes conformational rearrangement to support its NTase (nucleotidyltransferase) activity (105, 106). Like cGAS, OAS1 uses a conserved catalytic triad of residues (Asp75/Asp77/Asp148) that coordinate with two Mg^2+^ ions to catalytically add the donor ATP to the acceptor ATP or 2–5A (107). 2–5As, which function as a group of second messengers, can directly bind to RNase L and facilitate the assembly of an active form of the RNase L homodimer (**Figure 4**). Activated RNase L preferentially cleaves the UN^N (where ^ denotes the cleavage site) motif within ssRNA, attenuating protein synthesis and inducing apoptosis (108).

Compared to that of other RNA sensors, a unique property of OAS1 is that the C-terminal region of the human *OAS1* gene is alternatively spliced to produce the different isoforms p42, p46, p48 and p52, which are named according to their protein molecular weight (109). All these isoforms contain the complete domain architectures required for dsRNA recognition and NTase activity, but they exhibit distinct antiviral activities; for example, p42 and p46 have greater antiviral activity against Dengue virus than other isoforms (110). In addition, recent evidence has demonstrated that p46, which is not shared with other OAS1 isoforms, can suppress SARS-CoV-2 in cultured cells (27). Further analysis revealed that susceptibility to severe COVID-19 is associated with a SNP (single-nucleotide polymorphism) in the *OAS1* gene, which results in the *OAS1* isoform being expressed as p42 or p46. The molecular determinant of p46-specific anti-SARS-CoV-2 activity was subsequently mapped to a “CAAX” tail that is exclusive to p46. CAAX, a distinct four-amino-acid motif, is a signal for protein prenylation. This posttranslational modification is sufficient for driving proteins to the membrane, and in the case of p46, prenylation is harnessed to target OAS1 to perinuclear structures where the viral dsRNA is considered to be abundant (27).

The discovery that a SNP (rs10774671) in *OAS1* can exacerbate COVID-19 highlights the important role of genetic variations in *OAS1* in influencing disease risk (111). Likewise, genetic analysis of COVID-19 patients revealed that two other variants of *OAS1* result in a low expression level of the functional p46 isoform and dramatically increase the risk for severe COVID-19 (112). In addition to COVID-19, OAS1 variations and autoinflammatory immunodeficiency were recently correlated in two other reports (28, 113). Taken together, these findings demonstrated that, in addition to antiviral immunity, the OAS-RNase L pathway is crucial for the regulation of immune homeostasis, and genetic variations in OAS-related genes provide a new foundation for explaining disease susceptibility. However, functional and clinical insights into the basis of the genetic association between the OAS-RNase L axis and diseases are still under heated investigation.

## PKR signaling pathway

**Figure 5:**
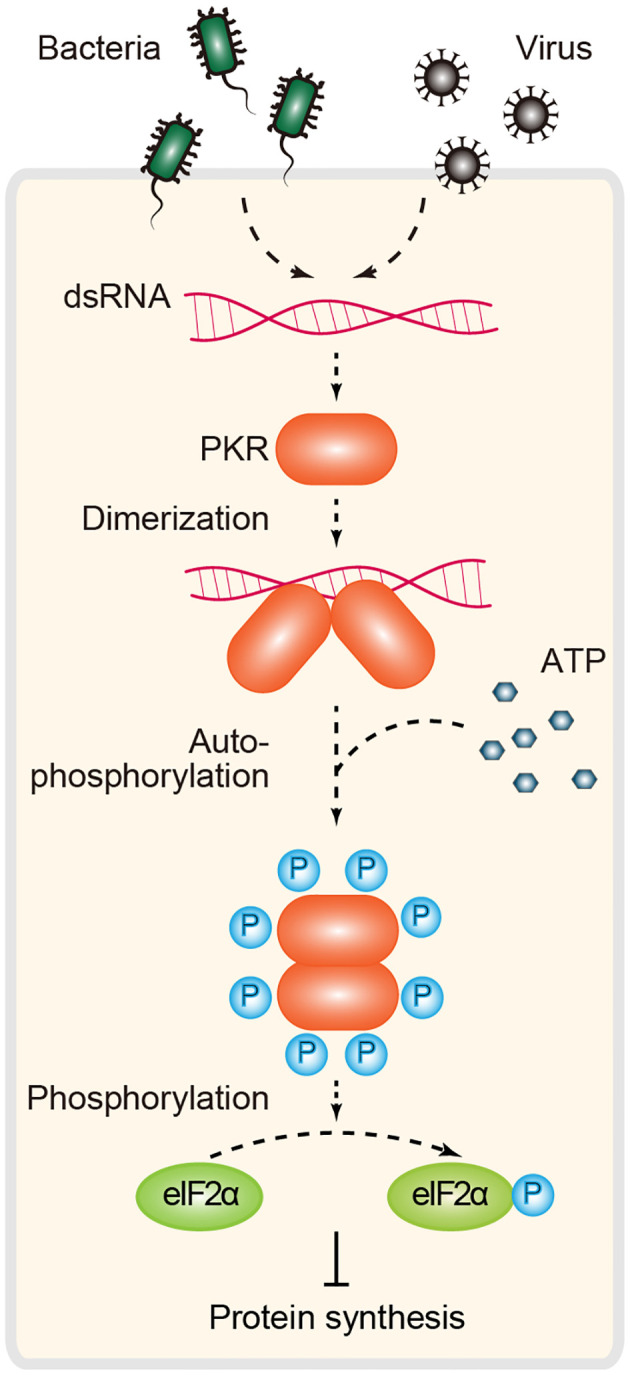
PKR-dependent immune signaling for RNA sensing. Inactive PKR behaves as a monomer. Binding of dsRNA to this protein kinase induces dimerization and promotes autophosphorylation. Active PKR subsequently inhibits translation initiation through phosphorylation of the α subunit of the initiation factor eIF2 (eIF2α). PKR, protein kinase R.

PKR is a dsRNA-dependent serine-threonine kinase with two N-term tandem dsRBMs (dsRNA-binding motifs) and a C-term kinase domain. A defining feature of PKR signaling is that activation is dependent on dsRNA length, and a dsRNA length ≥30 bp is required for PKR dimerization and efficient enzymatic activation (32). In the absence of agonist RNA, PKR is kept in an inactive form. Upon binding to dsRNA, PKR undergoes dimerization and autophosphorylation and transits to an active kinase, which is required for phosphorylating eIF2α (eukaryotic initiation factor-2α). Phosphorylated eIF2α inhibits cap-dependent translational initiation and arrests protein synthesis, resulting in the inhibition of cell growth and viral replication (4, 114, 115) (**Figure 5**).

In addition to the well-characterized translation inhibition mechanism, an alternative PKR strategy involves the regulation of MDA5-dependent IFN signaling. Growing evidence suggests that PKR promotes IFN induction in a manner independent of eIF2α inhibition under some conditions of viral infection (4). Mechanistically, it was proposed that PKR directly interacts with MAVS and is involved in MDA5-mediated IFN production and antiviral immunity (116, 117). PKR was originally identified as a restriction factor against viral infection. Building on this canonical model of PKR in antiviral immunity, there is also growing evidence that PKR acts as a multifunctional regulator involved in cell proliferation, apoptosis, metabolism, cancer and brain function, and the *EIF2AK2* (encoding PKR) missense mutant is associated with neurodevelopmental syndrome (4, 33, 115). This is probably because of the complexity of its upstream and downstream signals and because of its expression patterns at different developmental stages and cellular compartments. To date, several questions regarding the noncanonical functions (beyond antiviral immunity) of PKR have not yet been answered.

It has been proposed that short structured RNAs and inhibitory proteins can tightly bind and sequester PKR and prevent PKR activation (118, 119). As such, short dsRNA can induce PKR to adopt an alternative nonactivating dimer configuration, providing an additional mechanism for length-dependent RNA recognition and immune surveillance (120). TRBP (TAR RNA binding protein) is a direct inhibitor of PKR. TRBP has three dsRBMs, of which the first two are responsible for dsRNA binding, and the third mediates protein–protein interactions. Evidence in the field supports two potential models of PKR inhibition. One is that TRBP indirectly inhibits PKR through dynamic outcompeting of PKR for dsRNA binding, hence prohibiting the formation of PKR–dsRNA complexes that are essential for PKR enzymatic activation (121). In addition, TRBP directly interacts with PKR or the protein activator of PKR (PACT) to form heterodimers, thus inhibiting PKR activation (122). Interestingly, the positive regulator PACT shares domain similarity with TRBP, both of which contain three dsRBMs (123). However, compared to those of TRBP, the first two dsRBMs of PACT are responsible for PKR binding, and the third dsRBM is a unique PKR activation motif that may bind to an undefined site of PKR and contribute to PKR conformational change and activation (124). PKR signaling is an essential cellular mechanism limiting the spread of some viruses, and in turn, viruses have developed many evasion strategies through interfering with almost every step of PKR activation. For example, OV20.0, a protein of the orf virus, has been found to suppress PKR in three ways: binding to dsRNA to outcompete PKR for RNA binding; binding to the dsRBMs of PKR to release the association between PKR and PACT; and directly binding to PACT to inhibit PKR activation (125). Similarly, US11 of herpes simplex virus and NS1 of influenza A virus also directly bind to PKR to inhibit PKR signaling activation (126, 127). Taken together, these studies highlight conserved strategies for viral evasion and provide paradigms of the host–pathogen arms race.

## NOD-like receptors NLRP1 and NLRP6: sensing, signaling and evolution

**Figure 6:**
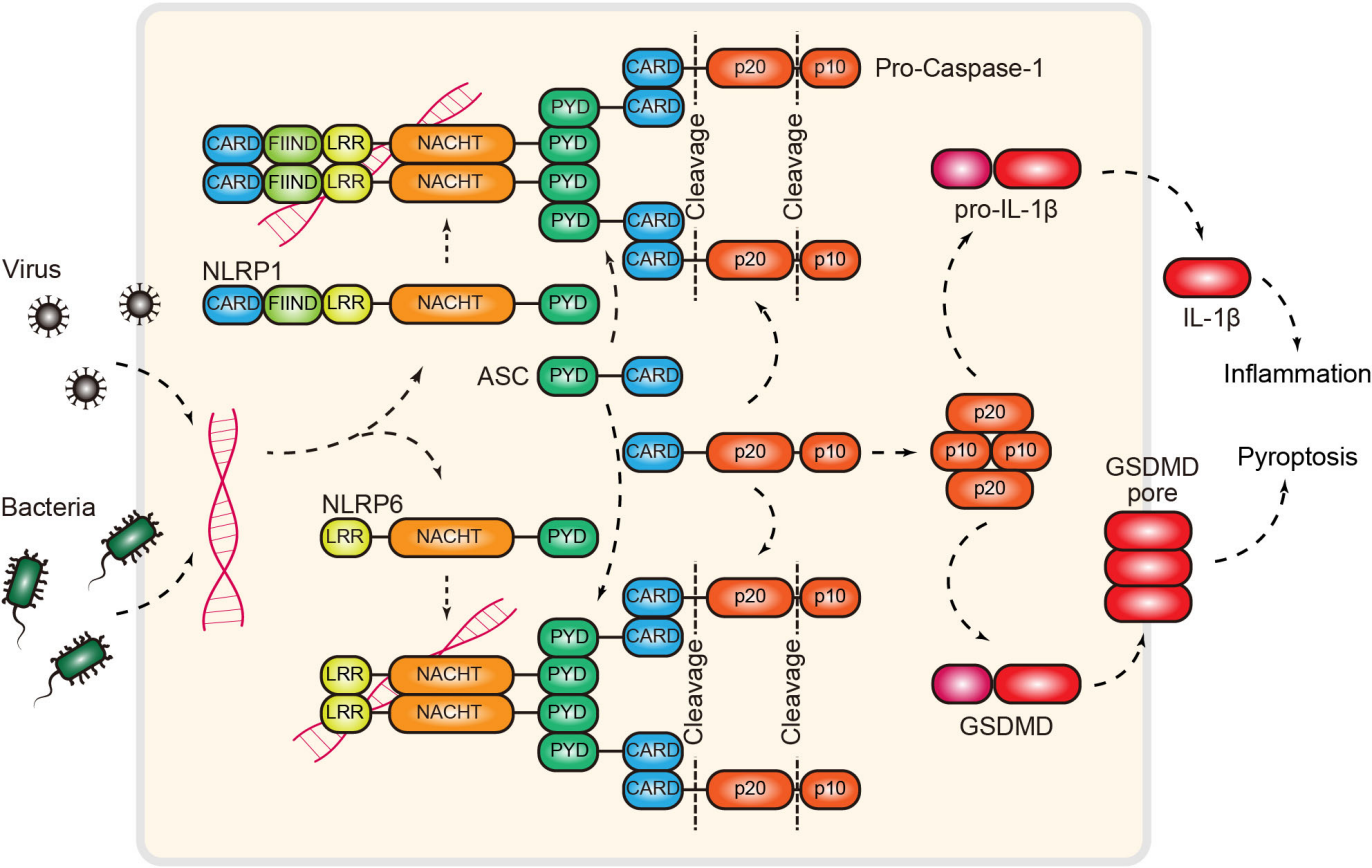
RNA-induced NLRP1/6 inflammasome. Inflammasomes can function as RNA sensors of pathogen infection. Mechanistically, NLRP1 and NLRP6, two NOD-like receptors, directly bind dsRNA and recruit the adaptor protein ASC via homotypic interactions. Next, ASC recruits pro-caspase-1 via the CARD domain. This interaction releases the autoinhibition of the protease activity of caspase 1. Activated caspase 1 then cleaves pro-IL-1β or gasdermin to induce inflammation or proptosis. NLRP1/6, NOD-like receptor family pyrin domain containing 1/6; CARD, caspase recruitment domain; FIIND, function-to-find domain; LRR, leucine-rich repeat; PYD, PYRIN domain; ASC, apoptosis-associated speck-like protein; GSDMD, gasdermin D; IL-1β, interleukin 1 beta.

NLRs (nucleotide-binding oligomerization domain (NOD)-like receptors) constitute the largest PRR family in animals, whereas 22 NOD-encoding genes have been identified in humans (128). NLRs typically sense danger signals and assemble into higher-order multiprotein complexes known as inflammasomes (**Figure 6**). Inflammasomes, which function as central networks of cellular machines, play essential roles in immune homeostasis (128). In fact, the NLR was first identified in plants, and these plant NLRs are the largest group of plant disease resistance (R) proteins (129). Remarkably, recent evidence has demonstrated that NLRs are widespread in bacteria and provide immunity against both DNA and RNA phages (130). In addition to NLRs, gasdermins, the key components in inflammasome signaling, were also recently characterized in bacteria (131). Identification of ancient NLRs and gasdermins in bacteria suggested that many proteins involved in NLR signaling first evolved in prokaryotes as antiphage defense systems. These new studies led to the discovery that NLR signaling is a broadly conserved mechanism across divergent kingdoms of life that enables cells to detect diverse dangers to induce downstream immune responses. However, the activating ligands of many NLRs have not yet been identified, and the downstream signaling pathways involved are largely unknown. Recent advances in the field of NLRs are discussed below.

NLRP1 and NLRP6 belong to the PYD (pyrin domain)-containing subgroup of NLRs (128) (**Figure 6**). PYD is the fundamental motif that enables the formation of ASC (apoptotic-associated speck-like protein) filaments through PYD-PYD interactions. ASC subsequently functions as a scaffold to recruit pro-caspase-1 via CARD interactions, resulting in the formation of a functional NLRP1/NLRP6 inflammasome (132). These complexes provide a foundation for the maturation of caspase-1 through autoproteolytic cleavage, followed by the activation of inflammation and pyroptosis (132, 133) (**Figure 6**). While this model provides a picture of how the NLRP1/6 inflammasome is assembled, signaling, and functioning, significant gaps remain. The most striking knowledge gap is what the activating ligands for these two NLRs are. To this end, two recent reports provided biochemical evidence that dsRNA is the missing cue for both NLRP1 and NLRP6 (34, 35). The Hornung laboratory showed that NLRP1 can directly bind dsRNA (>500 bp) via its LRR domain, resulting in the activation of ATPase activity in the NACHT domain (34). Similarly, building on previous findings, the Wu laboratory demonstrated that NLRP6 is a dsRNA sensor that restricts viral and bacterial infection (35, 134). Interestingly, they found that dsRNA induces NLRP6 to undergo LLPS (liquid–liquid phase separation) both *in vitro* and in cells (35). LLPS has recently emerged as an important mechanism that controls innate immune signaling (135, 136) (see the “cGAS” section). The discovery of NLRP-dsRNA phase separation raises several open questions; for example, how LLPS contributes to the formation of inflammasomes and whether LLPS is a common mechanism in NLR signaling, as liquid condensates exhibit biochemical properties distinct from those of canonical inflammasome filaments (137).

## Z-RNA sensing by ZBP1

The sensors discussed above detect the most common form of dsRNA molecules, named A-form RNAs, which adopt a right-handed conformation and are normally present at physiological salt concentrations. Notably, biochemical studies have demonstrated that some regions within A-form RNA may adopt a left-handed double helical structure, termed Z-form RNA or Z-RNA (138). A defining feature of Z-RNA is its relative instability due to its higher energy than A-RNA. Thus, Z-RNA requires stabilization by high salt concentrations or protein binding, and theoretically, protein binding is responsible for Z-RNA stabilization *in vivo*. Indeed, of the many proteins recognizing RNAs, several, such as ADAR1 and ZBP1 (Z-DNA-binding protein 1), specifically bind the unique left-handed helix Z-form of RNA in a structure-specific manner (57, 139). In particular, these proteins use a similar winged helix Zα domain to recognize and stabilize Z-RNA or Z-DNA (139).

ZBP1, originally named DLM-1, was initially discovered as a kind of tumor-related protein that functions in the host response to neoplasia (140). Identification of the Zα domain in ZBP1 led to the discovery that ZBP1 is a DNA sensor involved in innate inflammatory responses (141, 142). Characterization of the RHIM (RIP homotypic interaction motif) domain further linked ZBP1 with apoptotic and necroptotic cell death pathways (143–146). It is now known that ZBP1 senses unique Z-form structures, which are produced by a number of viruses, such as herpesvirus, orthomyxovirus and flaviviruses, and triggers different forms of cell death, including pyroptosis, apoptosis and necroptosis (collectively named PANoptosis) (36, 139, 145, 147). Once activated upon Z-RNA binding, ZBP1 interacts with the kinase RIPK3 via RHIM–RHIM interactions. Activated RIPK3 then phosphorylates and activates MLKL (mixed lineage kinase domain-like), triggering the execution of necroptosis (148, 149). The ZBP1–RIPK3 interaction can also trigger the activation of NLRP3 inflammasome, promote the release of proinflammatory cytokines and activate pyroptosis (145). Alternatively, ZBP1 activation is associated with the formation of the RIPK1–FADD–CASP8 complex and leads to apoptosis activation (150).

ADAR1, another Z-RNA-binding protein, has been shown to play a role in the regulation of ZBP1 signaling through the shielding of Z-RNA, preventing ZBP1 activation (151–153). Patient mutations occurring in the Zα domain of *ADAR1* have been associated with autoinflammatory pathology due to inappropriate activation of ZBP1 (154). Additionally, it was recently reported that ZBP1 can sense mitochondrial genome instability and induce the formation of the ZBP1–cGAS–RIPK1–RIPK3 complex to promote STAT1 phosphorylation, sustain IFN expression and drive cardiotoxicity (37, 155). Together, new advances in the field of ZBP1 have revealed that the use of Z-RNAs as immunostimulants to link cell death with host defense is an important mechanism of human antiviral immunity. However, due to technical limitations, the existence of Z-RNA and its dynamics *in vivo* have been confirmed only indirectly. Therefore, the following questions remain unanswered: What region in cellular RNA adopts a Z form? Does ZBP1 actually bind to Z-RNA in cells? Is ZBP1–Z-RNA binding extremely necessary for the activation of downstream immune signaling and cell death?

## cGAS-like receptors in RNA sensing

**Figure 7:**
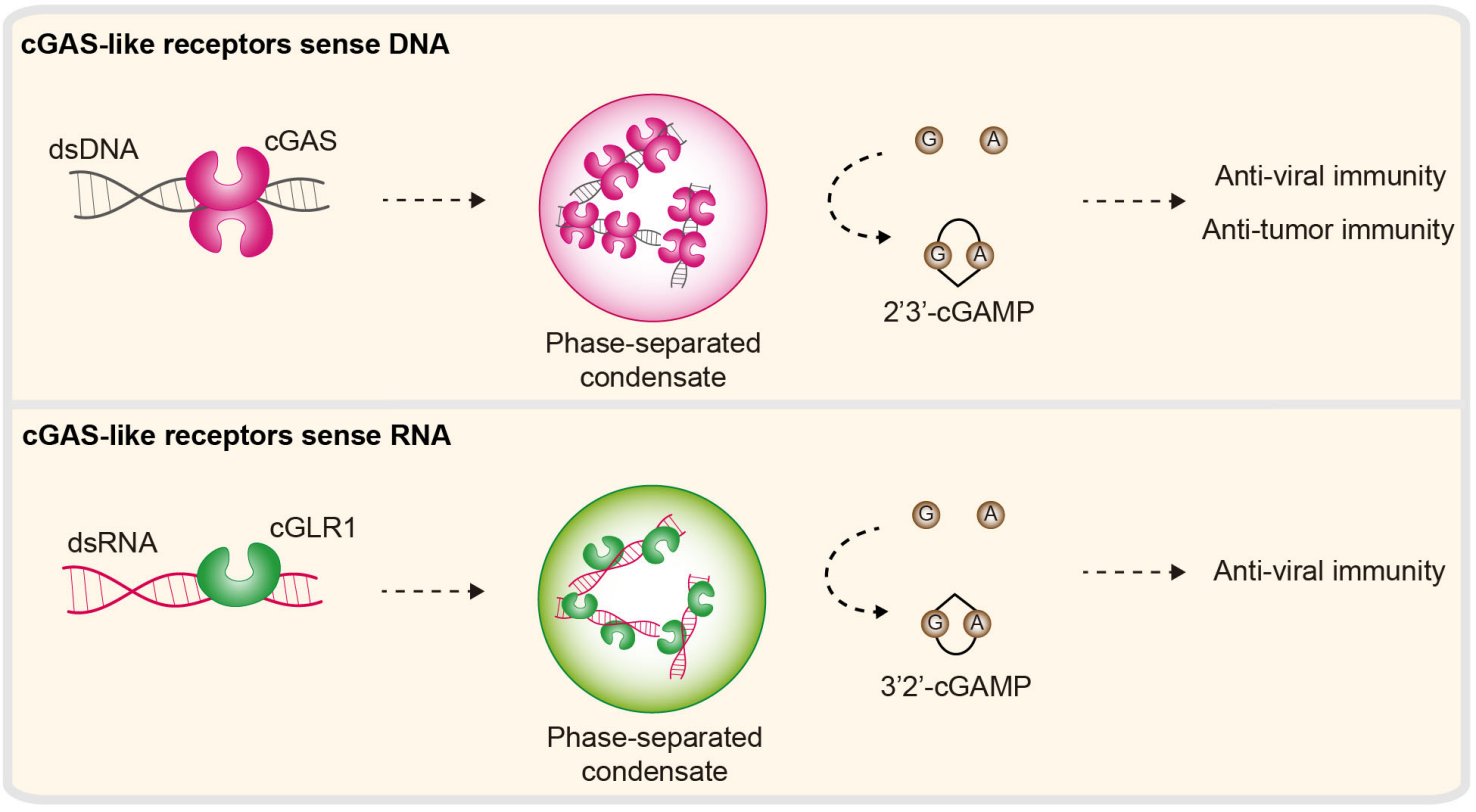
Nucleic acid sensing in cGLR immunity. cGAS-like receptors (cGLRs) are immune sensors that control cellular responses to DNA or RNA. Second, the enzyme cGAS is activated by DNA to synthesize 2′3′-cGAMP. DNA can further induce the formation of cGAS–DNA condensates. 2′3′-cGAMP functions as a second messenger and subsequently triggers antiviral and antitumor signaling programs. cGLR1, a homolog of cGAS, is a dsRNA sensor in *Drosophila melanogaster*. Activated cGLR1 synthesizes a chemical isomer of 2′3′-cGAMP, named 3′2′-cGAMP. 3′2′-cGAMP is a signal that triggers downstream antiviral immunity.

cGAS is an immune sensor in animals that controls the cellular response to dsDNA (156, 157). Upon dsDNA binding, cGAS undergoes conformational changes, which further allow the activation of its NTase activity to produce the nucleotide second messenger 2′3′-cGAMP. 2′3′-cGAMP is a small RNA molecule that is then recognized by the adaptor protein STING (stimulator of interferon genes) and ultimately generates type I IFNs and an NF-κB-dependent antiviral signaling program. Biochemical and structural studies reveal the details of how cGAS senses dsDNA and triggers immune signaling (158–160). The first step in cGAS activation is the direct interaction between dsDNA and a conserved ligand binding groove along the spine helix in cGLRs (cGAS-like receptors) (161). A Zn-ribbon motif, which is conserved in vertebrate cGLRs, is directly inserted into the dsDNA groove to control ligand specificity. DNA binding induces cGAS conformational changes and further supports the synthesis of nucleotide second messengers. Recent studies revealed that, beyond the fundamental requirement for cGAS activation, additional DNA-binding surfaces provide multivalent cGAS–DNA interactions that further drive the formation of biomolecular condensates via a mechanism named LLPS (135, 136, 162) (**Figure 7**). Further evidence has demonstrated that LLPS of cGAS is not necessary for its intrinsic enzymatic activity *in vitro*; rather, it is necessary to ensure potent immune activation in the cellular environment via negative regulators (e.g., TREX1 and BAF) (136, 163).

It is well known and accepted that cGAS is a dsDNA sensor involved in immune responses. However, this concept has been called into question very recently by the Kranzusch Lab and others. They concluded that human cGAS signaling is evolutionarily conserved across the tree of life, and most importantly, several cGLRs across various life forms are exclusively activated by dsRNA (41, 54, 164). The best characterized of these, *Drosophila melanogaster* cGLR1 (cGAS-like receptor 1), is a dsRNA sensor (39). Upon dsRNA binding, cGLR1 synthesizes 3′2′-cGAMP (an analog of 2′3′-cGAMP) and activates downstream STING-dependent antiviral immunity (39, 40) (**Figure 7**). Similarly, a recent report revealed that the bacterium cGLR (e.g., CdnE03 cyclase) can be activated by cabRNA (CBASS-activating bacteriophage RNA), which is a structured RNA transcribed from terminase subunit genes during phage infection. It was proposed that the secondary/tertiary structure is essential for the activation of the bacteria cGLR (42). Intriguingly, these dsRNA-dependent cGLRs exhibit high biochemical and structural similarity with cGAS, including structural and domain architectures, positively charged spine helices and NTase activity. Therefore, how cGLRs have evolved to sense and discriminate DNA and RNA is unknown.

## Conclusions and future perspectives

The use of immune sensors to detect pathogens is a conserved mechanism of antiviral immunity shared between prokaryotes and eukaryotes. Importantly, many sensors and signaling components that are considered exclusively vertebrate proteins have now been identified in bacteria, demonstrating that the core machinery of RNA-sensing immunity is shared across the tree of life. Linked to this point, how species-specific adaptations drive specificity and selectivity in immune responses to RNA remains an important area of investigation. In addition to distinct classes of nucleic acid sensors, emerging evidence suggests that many RNA- and DNA-sensing pathways are physically and functionally interconnected; for example, the cytosolic RIG-I-MAVS and cGAS-STING nucleic acid-sensing pathways coordinately amplify the innate antiviral responses against both RNA and DNA viral pathogens (165). In another example of crosstalk signaling, ZBP1 forms a complex with cGAS and RIPKs to sustain type I IFN signaling and cooperatively drive pathogenesis (37, 155). Further elucidation of the biochemical, structural, and mechanistic details of these crosstalk events will be essential for revealing their functional importance and for developing new rational strategies for the treatment of infection, autoimmunity and cancer.

An important feature of RNA sensors is their ability to discriminate self from nonself, which is thought to be an evolutionary advantage in maintaining immune homeostasis. However, the striking discovery that endogenous RNAs are native ligands for almost all known RNA sensors highlights the expanded role of RNA-sensing pathways in RNA biology. An open question is whether, for a given sensor, the immune response to self and nonself is achieved together from the same components or alternatively through distinct mechanisms. Recent analysis revealed that LLPS is a novel mechanism controlling the innate immune response to RNA. In the NLRP6 pathway, dsRNA can induce NLRP6 to underdo LLPS and promote innate antiviral immunity (35). A further question is whether LLPS is a common regulatory mechanism in RNA sensing. In addition, whether there is an opportunity to improve the understanding of RNA sensing in the therapeutic design of novel treatments for infectious diseases, autoimmune disorders and cancer is worthy of future investigation. Importantly, the current knowledge of RNA sensors and the underlying mechanisms of signaling activation provides fundamental information for designing RNA vaccines with efficiency and safety. In particular, the understanding of self- and nonself- related mechanisms has led to the development of RNA modification as a key strategy for RNA vaccines to lower overall immunogenicity and increase RNA stability and translation efficiency. A comprehensive discussion of RNA vaccines is now available in another Review article of the same Topic section (166).

## Author contributions

XL: Writing – original draft. LW: Conceptualization, Data curation, Formal analysis, Visualization, Writing – original draft, Writing – review & editing. GS: Data curation, Writing – review & editing. WZ: Conceptualization, Data curation, Formal analysis, Funding acquisition, Resources, Supervision, Visualization, Writing – original draft, Writing – review & editing.
